# Facile in Situ Transformation of NiOOH into MOF-74(Ni)/NiO OH Heterogeneous Composite for Enchancing Electrocatalytic Methanol Oxidation

**DOI:** 10.3390/molecules27072113

**Published:** 2022-03-25

**Authors:** Wei-Qun Zhou, Ben-Jun Xi, Xi-Wen Chang, Bin Wang, Xue-Qian Wu, Shuang Li, Ya-Pan Wu, Dong-Sheng Li

**Affiliations:** 1 Key Laboratory of Inorganic Nonmetallic Crystalline and Energy Conversion Materials, College of Materials and Chemical Engineering, China Three Gorges University, Yichang 443002, China; zhouweiqunmail@163.com (W.-Q.Z.); changxiwen666@163.com (X.-W.C.); wuxueqiansnail@163.com (X.-Q.W.); lishmail@126.com (S.L.); 2 Hubei Three Gorges Laboratory, Yichang 443007, China; xibenjun@xingfagroup.com (B.-J.X.); congratulationswangbin@163.com (B.W.)

**Keywords:** nickel oxide hydroxide, MOF-74(Ni)/NiOOH composite, in situ, electrocatalysis, methanol oxidation reaction

## Abstract

A new MOF-74(Ni)/NiOOH heterogeneous composite was synthesized via NiOOH microsphere precursor. The electrocatalytic methanol oxidation reactions’ (MOR) performance was assessed. The as-prepared MOF-74(Ni)/NiOOH exhibited excellent activity with high peak current density (27.62 mA·cm^−2^) and high mass activity (243.8 mA·mg^−1^). The enhanced activity could be a result of the synergistic effect of the MOF-74(Ni)/NiOOH heterocomposite providing more exposed active sites, a beneficial diffusion path between the catalyst surface and electrolyte, and improved conductivity, favorable for improving MOR performance.

## 1. Introduction

Nowadays, the increasing consumption of nonrenewable fossil resources and triggered environmental deterioration have motivated extensive enthusiasm for clean energy research green, clean and sustainable energy storage, and technology transformation [1,2,3,4]. Fuel cells have good potential and excellent development prospect owing to environmental friendliness, high energy density, and power generation efficiency. In particular, the direct methanol fuel cell (DMFC) is becoming one of the most ideal alternative technologies in the near future due to its advantages of low cost, simple structure, high efficiency, etc. [5,6,7]. However, the efficiency is severely hampered by the methanol oxidation reaction (MOR) related to multistep electron transfer, thus leading to sluggish reaction kinetics [8]. Currently, noble metal Pt/Ru/Ir nanomaterials and their alloys are the most substantial electrocatalysts to date, even so, their low abundance, high cost, and easy surface poisoning through adsorbed intermediates limit the industrial application [9,10,11]. Therefore, it is of great importance to seek alternative non-noble metal-based catalysts with the merits of low-cost and high MOR performance.

Nickel oxide hydroxide (NiOOH), with a unique layered structure, has promising electrocatalytic activities for a series of important electrocatalytic processes such as the oxygen evolution reaction, alcohol oxidation, and the urea oxidation reaction [12,13,14]. The unique staggered structure provides a channel for electron transfer and chemical species’ exchange between electrolyte and electrocatalyst. This catalyst, however, still has high overpotential and poor long-term stability [15,16]. Therefore, we should develop the appropriate synthetic strategy to design novel and efficient electrocatalysts, which can not only utilize the advantage of NiOOH but also improve its catalytic activity in electrochemical oxidation reactions.

Metal-organic frameworks (MOFs) have been extensively studied in electrochemical energy applications due to the intrinsic structural advantages of high surface area, inherently present open metal sites, tunable porosity, and heteroatom doping [17,18,19]. Importantly, these materials could be rationally designed and embellished at a molecular level to achieve the specific electrochemical application by selecting redox-active metal ions and organic ligands [20]. Nevertheless, the fragile coordination chemical bonding between metal ions and functional site of organic ligands and poor conductivity are still hindering their broader applications in electrocatalysis. To further improve the electrocatalytic activities of MOF catalysts, various post-treatment strategies, such as high temperature annealing, solvothermal routes, and integration with other components, were explored recently [21,22]. Among them, sacrificing target materials with unique nanostructures might be another choice to tap the potential of MOFs for the electrocatalytic process [23,24]. As a classical and important sort of metal hydroxides, NiOOH are capable of delamination and restacking with exchangeable positive ions and interlayer charge-balancing anions, However, MOF nanostructures, especially for MOFs transformed from NiOOH, would afford us efficient performance with energy, but have not been demonstrated so far.

The well-known MOF-74 family has been confirmed as a microporous material, which can be used as an electrocatalytic electrode to accelerate the ingression and contact between electrolyte and catalyst and facilitate the catalytic reaction process. Inspired by the above-mentioned points, we proposed a simple in situ transformation method to fabricate an MOF-74(Ni)/NiOOH heterogeneous composite by employing solvothermally obtained NiOOH microspheres as the precursor. The larger active surface area and better charge-transfer property of the MOF-74(Ni)/NiOOH heterostructure were considered to promote the highly active and stable electrocatalytic properties toward MOR in an alkaline electrolyte. As expected, the as-synthesized MOF-74(Ni)/NiOOH sample delivered enhancing electrocatalytic MOR performance with high peak current density (27.62 mA·cm^−2^) and high mass activity (243.8 mA·mg^−1^). This facile and steerable in situ preparation of the MOF-74(Ni)/NiOOH heterogeneous composite from a NiOOH microsphere precursor can provide better electrocatalysts for practical applications.

## 2. Results and Discussion

**Figure 1:**
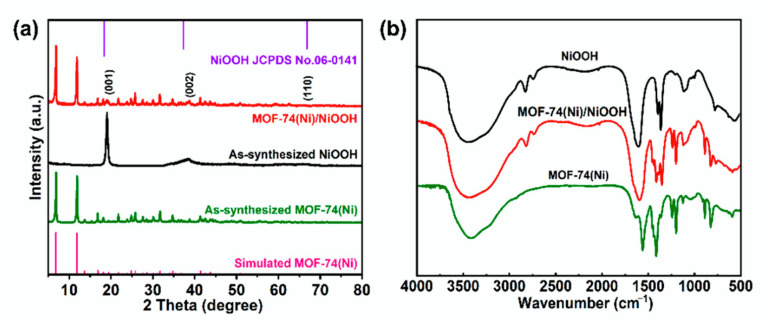
(**a**) PXRD patterns of the as-prepared NiOOH, MOF-74(Ni), and MOF-74(Ni)/NiOOH. (**b**) FT-IR patterns of as-prepared NiOOH, MOF-74(Ni), and MOF-74(Ni)/NiOOH samples.

The NiOOH precursor was prepared by a simple reaction between Ni(OH)_2_, K_2_S_2_O_8_, and NaOH solution at room temperature for 24 h. The MOF-74(Ni)/NiOOH heterostructure was prepared through a facile in situ semitransformation process of the NiOOH precursor. The features of MOF-74(Ni)/NiOOH and MOF-74(Ni) were characterized by PXRD and IR techniques. PXRD was tested to assess the phase transformation of the as-synthesized samples. As shown in Figure 1a, the key diffraction peaks of the NiOOH precursor could be indexed to pure NiOOH (PDF No. 06-0141) phase, in good agreement with previous reports for NiOOH. With the introduction of a 2,5-dihydroxyterephthalic acid (DOBDC) ligand under the same synthetic condition, some characteristic peaks of MOF-74(Ni) could be observed, which confirmed the successful generation of MOF-74(Ni) constructed from the DOBDC and NiOOH precursor. Moreover, the sharp absorption bands appearing at about 3485 cm^−1^ in the FTIR spectrum for MOF-74(Ni) (Figure 1b) were ascribed to the stretching vibrations of the O-H of adsorbed water molecules in MOF-74(Ni). Two bands centered at 1580 and 1375 cm^−1^ corresponded to the asymmetric and symmetric stretching modes of coordinated –COO^−^, respectively. The FT-IR spectrum of MOF-74(Ni)/NiOOH composite displayed similar characteristic absorption bands with MOF- 74(Ni), in particular, MOF-74(Ni)/NiOOH still retained the characteristic absorption peak of O-H at around 2850 cm^−1^(Figure 1b), indicating the successful in situ growth of MOF-74(Ni) on NiOOH.

**Figure 2:**
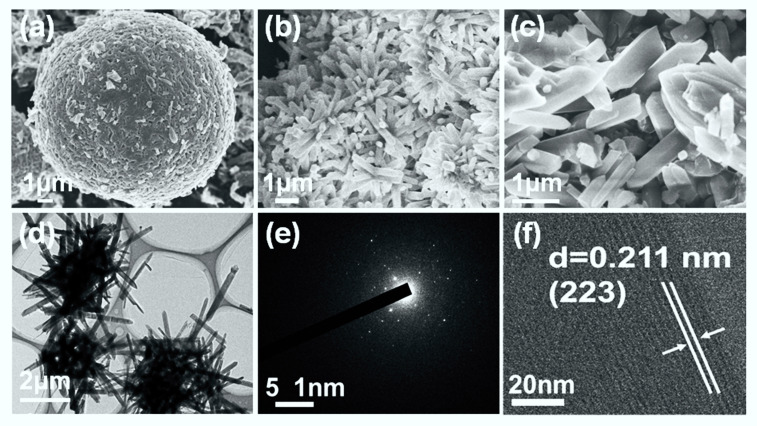
(**a**) SEM image of NiOOH. (**b,c**) SEM image of MOF-74(Ni)/NiOOH. (**d**) TEM image of MOF-74(Ni)/NiOOH, (**e**) SEAD pattern of MOF-74Ni/NiOOH. (**f**) HRTEM image of MOF-74Ni/NiOOH.

The corresponding morphological evolution of NiOOH and MOF-74(Ni)/NiOOH were characterized by field emission scanning electron microscopy (SEM). As shown in Figure 2a and Figure S1a, the obtained NiOOH precursor had a hierarchical microsphere structure self-assembled from small NiOOH sheets. When reacting with a specific amount of DOBDC ligand, the as-prepared MOF-74 (Ni)/NiOOH (Figure 2b,c and Figure S1b) showed aggregation from a large amount of nanorods. The spherical NiOOH was not observed due to its low content. Furthermore, a transmission electron microscopy (TEM) image further revealed the morphology of the nanorods for MOF-74(Ni)/NiOOH (Figure 2d). The selected electron diffraction (SAED) confirmed that MOF-74(Ni) particles had the nature of crystalline state (Figure 2e). High-resolution TEM (HRTEM) showed the lattice fringes of 0.211 nm, which might be ascribed to the (223) plane of MOF-74(Ni). The unique hierarchical structure originated from the intermediate status during the in situ semitransformation reaction of NiOOH. The NiOOH microballoons were destroyed, and MOF-74(Ni) was generated on the surface. This well-arranged hierarchical structure could provide rapid charge and mass transport of the electrolyte to the reaction sites of the catalyst surface, resulting in an improved electrocatalytic process.

**Figure 3:**
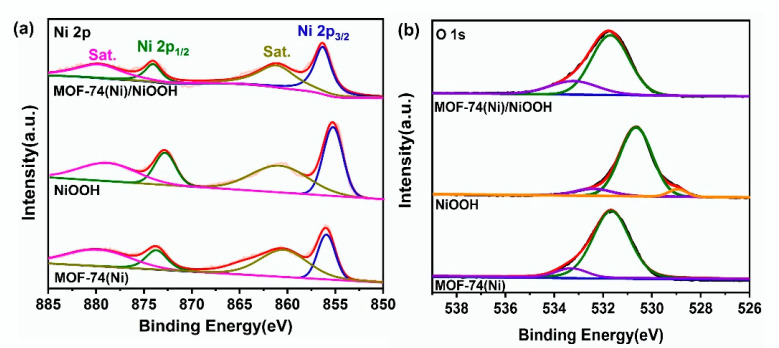
High-resolution XPS spectra of Ni2p (**a**) and O1s (**b**) of NiOOH, MOF-74(Ni), and MOF-74(Ni)/NiOOH.

Moreover, to understand the electronic interaction and the electronic state and chemical composition, the elements of Ni 2p and O 1s of NiOOH, MOF-74(Ni) and MOF-74(Ni)/NiOOH were characterized by X-ray photoelectron spectroscopy (XPS). The XPS full spectrums of NiOOH, MOF-74(Ni), and MOF-74(Ni)/NiOOH are shown in Figure S2. Obviously, the signal of element C was captured in MOF-74(Ni)/NiOOH (Figure S3). The high-resolution XPS spectra of the Ni 2p (Figure 3a) at around 856.2 and 874.0 eV could be defined as Ni 2p3/2 and Ni 2p1/2 electronic configurations, respectively. It was observed that the characteristic Ni2p3/2 and Ni 2p1/2 peaks for MOF-74(Ni)/NiOOH were close to that for NiOOH, but two Ni2p peaks were slightly moved to the higher binding energy location, confirming that the Ni ions in MOF-74(Ni)/NiOOH had lower electron densities than those of NiOOH. Additionally, in Figure 3b, the O1s spectrum for the composite of MOF-74(Ni)/NiOOH combined the characteristics of NiOOH and MOF-74(Ni), which may be resulted from the interaction between the two phases. The interaction between DOBDC ligands and Ni ions released from NiOOH modulated the electronic state of the NiOOH surface, which may have enhanced the catalytic activity for MOR. Consequently, better electrochemical MOR activities could be expected for MOF-74(Ni)/NiOOH.

**Figure 4:**
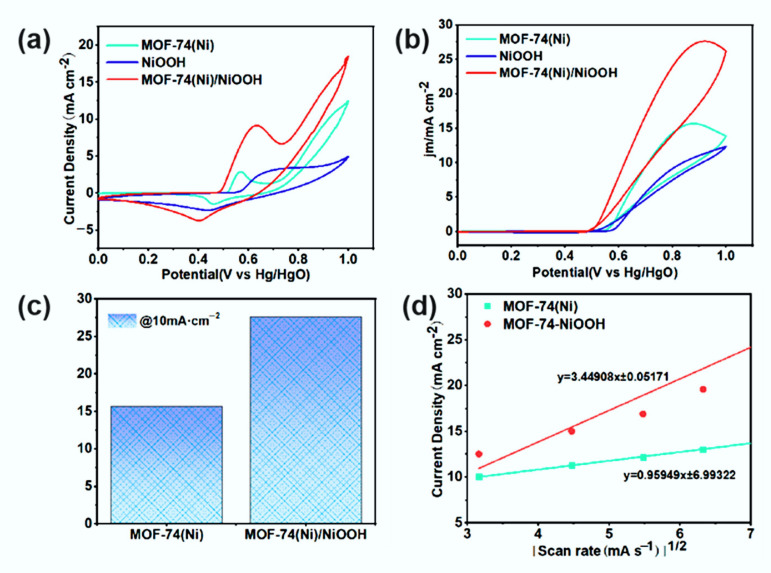
(**a**) Cyclic voltammetric responses of NiOOH, MOF-74(Ni), and MOF-74(Ni)/NiOOH catalysts. (**b**) MOR curves of different catalysts operated in 0.1 M KOH in the presence of 1.0 M CH_3_OH. (**c**) Comparison of peak current densities for different catalysts. (**d**) Linear relation between the current densities and square root of scan rates of MOF-74(Ni) and MOF-74(Ni)/NiOOH catalysts.

To study the catalytic activities of the as-prepared samples, electrocatalytic activities toward MOR of NiOOH, MOF-74(Ni), and MOF-74(Ni)/NiOOH were investigated in typical three-electrode 0.1 M KOH with and without 1.0 M methanol solution. All as-synthesized catalysts were coated on glassy carbon electrode (GCE) acting as the working electrode, and their current densities were used to evaluate the catalytic activity of the material. As shown in Figure 4a, the CV curves of NiOOH, MOF-74(Ni), and MOF-74(Ni)/NiOOH in 0.1 M KOH solution at a scan rate of 50 mV s^−1^. Apparently, the peaks to Ni^III^ were anodic and cathodic at around 0.55 and 0.65 V, respectively, which should be attributed to the conversion of Ni^II^ during the catalyst activation. Moreover, the electrocatalytic MOR activity of three catalysts was tested in the solution of 0.1 M KOH in the presence of 1.0 M CH_3_OH (Figure 4b). All as-prepared samples showed markedly varied catalytic activity for methanol oxidation. Noteworthy was the markedly varied highest peak current densities activity (27.62 mA·cm^−2^) and mass activity (243.8 mA·mg^−1^), which was around 1.8 times higher than that of pure MOF-74(Ni) (15.65 mA·cm^−2^) and mass activity (138.3 mA·mg^−1^) (Figure 4c), indicating its improving catalytic activity for MOR, which could be attributed to the synergistic effect between MOF-74(Ni) and NiOOH. Moreover, the peak current densities of MOF-74(Ni) and MOF-74(Ni)/NiOOH increased linearly with the square root of the scan rate for sweep rates from 10 to 50 mV s^−1^, indicating that their MOR process was determined by the same diffusion speed (Figure 4d). The diffusion constant (D) was determined by the Randles–Sevcik equation: ip = 0.4463 nFAC(nFvD/RT)^1/2^ [25]. The diffusion coefficient values were 6.77 × 10^−8^ cm^2^·s^−1^ for MOF-74(Ni) and 2.11 × 10^−7^ cm^2^·s^−1^ for MOF-74(Ni)/NiOOH, respectively. The results indicated that the system fulfilled the diffusion-controlled mechanism. Apart from catalytic peak current densities, the amount of catalyst was another index to assess MOR performance. Quality-dependent cyclic voltammetry (CV) was carried out for MOF-74(Ni)/NiOOH with different amounts operated in 0.1 M KOH in the presence of 1.0 M CH_3_OH (Figure S5). When there was 6 μL of the catalyst, MOF-74(Ni)/NiOOH attained the highest peak current densities (27.62 mA·cm^−2^).

Furthermore, electrochemical impedance spectroscopy (ESI) was performed to investigate the electrode kinetics of NiOOH, MOF-74(Ni), and MOF-74(Ni)/NiOOH. As shown in Figure S4, the EIS Nyquist curves for the catalysts were obtained at onset potentials. By contrast, the Rct value of MOF-74(Ni)/NiOOH (216 Ω) was smaller than those of the other three samples, also confirming that MOF-74(Ni)/NiOOH could be beneficial to the fast electron transport efficiency and improved MOR kinetics. The stability was another significant criterion to assess the performance of an electrocatalyst. Thus, the chronoamperometry test was conducted to evaluate the durability of the samples in 0.1 M KOH-containing 1.0 M CH_3_OH solution. As can be seen, the current almost kept no obvious changes over 3600 s for the MOF-74(Ni)/ NiOOH sample (Figure S4 inset), which could be a benefit from the coupling effect between MOF-74(Ni) norods and the NiOOH microballoon with excellent MOR activities.

**Figure 5:**
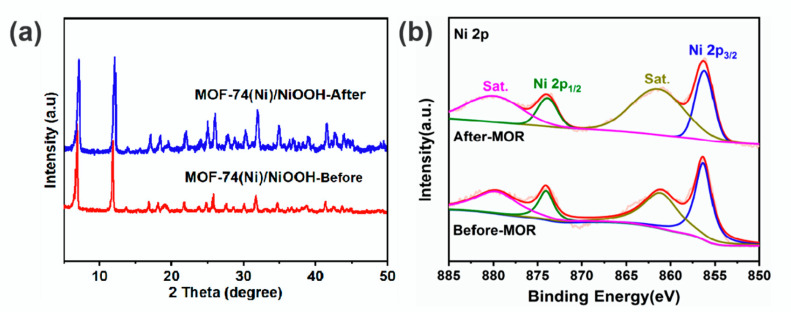
(**a**) PXRD pattern of MOF-74(Ni)/NiOOH catalysts before and after the MOR process. (**b**) High-resolution XPS spectra of Ni2p of MOF-74(Ni)/NiOOH catalyst before and after MOR.

Additionally, to confirm the microstructure of MOF-74(Ni)/NiOOH after MOR, some characterizations including PXRD, XPS, and SEM techniques also confirmed the persistence of MOF-74(Ni)/NiOOH after MOR tests. PXRD curves recorded after MOR indicated that the MOF-74(Ni)/NiOOH sample was stable (Figure 5a). The XPS spectra of MOF-74(Ni)/NiOOH before and after MOR were also assessed (Figure S6). The high-resolution XPS spectra of Ni2p recorded both before and after catalysis showed two characteristic peaks with binding energies of about 874.0 and 856.2 eV (Figure 5b). There were no significant changes in the Ni2p peaks before and after catalysis, which also revealed that the chemical and structural environment was retained. Meanwhile, the SEM measurements indicated that MOF-74(Ni)/NiOOH retained its morphology during the MOR process (Figure S7). On the whole, the above results undoubtedly confirmed that MOF-74(Ni)/NiOOH attained good MOR performance with vigorous durability.

## 3. Materials and Methods

### 3.1. Materials

2,5-dihydroxyterephthalic acid (H_2_DOBDC), nickel nitrate (Ni(NO_3_)_2_·6H_2_O, 99.9%), and nickel hydroxide (Ni(OH)_2_, 98%) were provided by Alfa Aesar. Other chemicals such as potassium peroxydisulfate (K_2_S_2_O_8_, 95%), sodium hydroxide (NaOH, >98%), N, N-dimethyl formamide (DMF), were purchased from Sinopharm Chemical Reagent Co. Ltd. All the chemicals were used as received without further purification. All aqueous solutions were performed in deionized water with a resistivity of 18.2 MΩ cm.

### 3.2. Preparation of Materials

#### 3.2.1. Preparation of NiOOH

A mixture of Ni(OH)_2_ (140 mg), K_2_S_2_O_8_ (600 mg), and 100 mL NaOH (1.0 M) was mixed and stirred at room temperature for 24 h. The fluid after the reaction was vacuumized and filtered. The product NiOOH was washed with deionized water and ethanol, respectively, and dried at 80 °C under vacuum overnight.

#### 3.2.2. Preparation of MOF-74(Ni)/NiOOH

In a 25 mL Teflon-lined stainless-steel vessel, 40 mg of 2,5-dihydroxyterephthalic acid and 56 mg of NiOOH were dispersed in 5 mL of dimethylformamide, 5 mL of ethanol, and 5 mL of water. The vial was tightly sealed and put into an oven at 120 °C for 24 h. After cooled to room temperature, the products were filtered and washed three times with DMF and methanol. The samples MOF-74(Ni)/NiOOH were heated under vacuum to 80 °C for 12 h.

#### 3.2.3. Preparation of MOF-74(Ni)

MOF-74(Ni) was prepared according to a slightly modified procedure from the literature [26]. The preparation process was the same as that of the MOF-74(Ni)/NiOOH, except that the as-prepared NiOOH was changed by the Ni(NO_3)2_·6H_2_O for MOF-74(Ni).

### 3.3. Characterizations

Powder X-ray diffraction (PXRD) was studied on a Rigaku Ultima IV diffractometer (Cu Kαradiation, λ = 1.5406 Å). FT-IR spectra (KBr pellets) were conducted on a Thermo Electron NEXUS 670 FTIR spectrometer. A field emission scanning electron microscope (FESEM, JSM-7500F, operating accelerating voltage of 20 kV) and a Tecnai G2F20 S-TWIN transmission electron microscope (TEM, 200 kV) were used to analyze the morphologies and structures of the as-synthesized samples. X-ray photoelectron spectrometry (XPS) was carried out on an ESCA LABMKLL X-ray photoelectron spectrometer using an AlKα source.

### 3.4. Electrochemical Measurements

Electrochemical measurements were carried out with computer-controlled potentials (CHI660E, CH instrument, Shanghai, China) with a three-electrode electrochemical cell containing aqueous solution as the electrolyte. The working electrode adopted a glassy carbon electrode (GCE, about 3 mm inner diameter, 0.0706 cm^2^), and Hg/HgO and Pt wire were used as the reference electrode and the counter electrode, respectively. The catalyst ink was made by dispersing 4 mg of the as-synthesized sample into 2 mL of ethanol, 0.8 mL of deionized water, and 0.2 mL of 5 wt% Nafion and sonicated for 30 min. Then, 6 μL of the catalyst (loading amount: ~0.008 mg) ink was coated onto a GCE surface and naturally dried at room temperature. The static cyclic voltammetry (CV) measurement was performed in the N_2_-saturated 0.1 M KOH electrolyte, and the properties of MOR were studied in the N_2_-saturated 0.1 M KOH + 1.0 M CH_3_OH electrolyte with a scan rate of 50 mV s^−1^. All the electrochemical data were directly converted without iR correction.

## 4. Conclusions

In summary, a well-defined heterostructure MOF-74(Ni)/NiOOH was prepared by in situ self-sacrificing template strategy. It should be a potent MOR catalyst in alkaline solutions, which can be attributed to the created unique heterostructure. The as-synthesized hybrid material MOF-74(Ni)/NiOOH showed higher electrocatalytic MOR activity compared to that of the pure MOF-74(Ni) catalyst. This work not only put forward an innovative strategy to construct a superior NiOOH-based heterogeneous composite, but also paved a new way for designing highly efficient Ni-based electrocatalysts for methanol oxidation in the field of energy conversion.

## Data Availability

Not applicable.

## Supplementary Materials

The following supporting information can be downloaded at: https://www.mdpi.com/article/10.3390/molecules27072113/s1, Figure S1. SEM image of NiOOH (a) and MOF-74(Ni)/NiOOH (b) with the same magnification; Figure S2. XPS survey spectrum of NiOOH, MOF-74(Ni) and MOF-74(Ni)/NiOOH; Figure S3. The high-resolution XPS spectra of C1s of MOF-74(Ni) and MOF-74(Ni)/NiOOH; Figure S4. Electrochemical impedance spectra (EIS) of different electrocatalysts. (Inset: Durability measurements for MOF-74(Ni)/NiOOH); Figure S5. MOR curves of MOF-74(Ni)/NiOOH with different amount operated in 0.1 mol·L-1KOH in presence of 1.0 mol·L^−1^ CH3OH; Figure S6. XPS survey spectrum of MOF-74(Ni)/NiOOH before and after MOR; Figure S7. SEM image of MOF-74(Ni)/NiOOH after MOR.

## Sample Availability

Samples of the compounds are available from the authors.
