# Supplementary material for: Facile in Situ Transformation of NiOOH into MOF-74(Ni)/NiO OH Heterogeneous Composite for Enchancing Electrocatalytic Methanol Oxidation
Source: Molecules. 2022 Mar 25;27(7):2113. doi: 10.3390/molecules27072113 (PMC9000767; doi:10.3390/molecules27072113)
Supplement: Supplementary file 1 [file molecules-27-02113-s001.zip › molecules-1617388-supplementary.pdf]

Supplementary Information

Facile in situ transformation of NiOOH into MOF-74(Ni)/NiOOH heterogeneous composite for enchancing electrocatalytic methanol oxidation

Wei-Qun Zhou [1,2], Ben-Jun Xi[2], Xi-Wen Chang[1,2,] Bin Wang[2], Xue-Qian Wu[1,2], Shuang Li[1,2], Ya-Pan Wu [1,2]* and Dong-Sheng Li[1, 2,]*

[1.] *College of Materials and Chemical Engineering, Key Laboratory of Inorganic Nonmetallic Crystalline and Energy Conversion Materials, China Three Gorges University, Yichang, 443002, China*

[2.] *Hubei Three Gorges Laboratory, Yichang, 443007, China*

* Correspondence: E-mail: wyapan2008@163.com(Y.P.Wu);
lidongsheng1@126.com(D.S. Li)

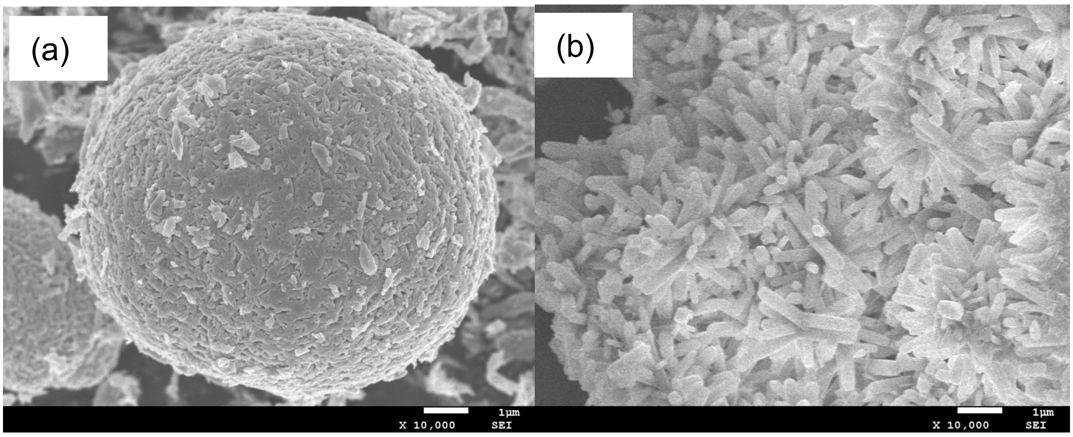

Figure S1. SEM image of NiOOH (a) and MOF-74(Ni)/NiOOH (b) with the same magnification.

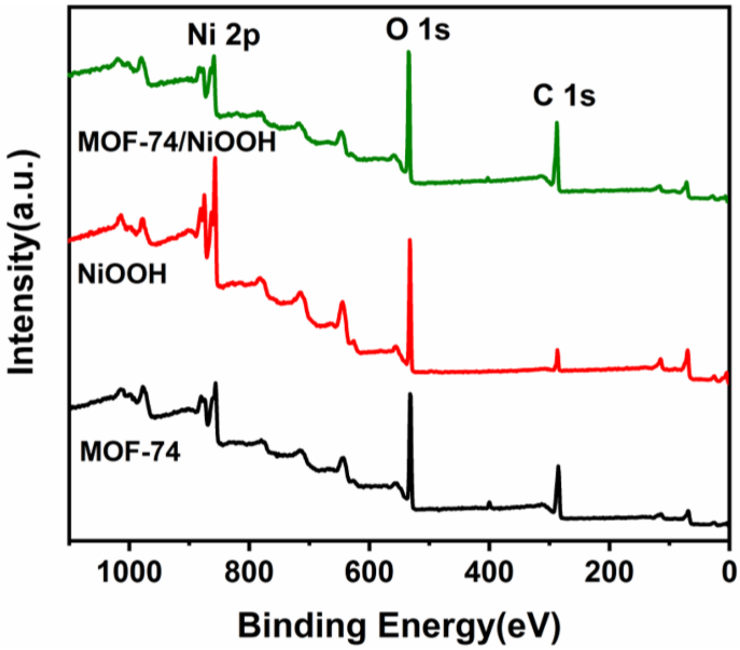

Figure S2. XPS survey spectrum of NiOOH, MOF-74(Ni) and MOF-74(Ni)/NiOOH.

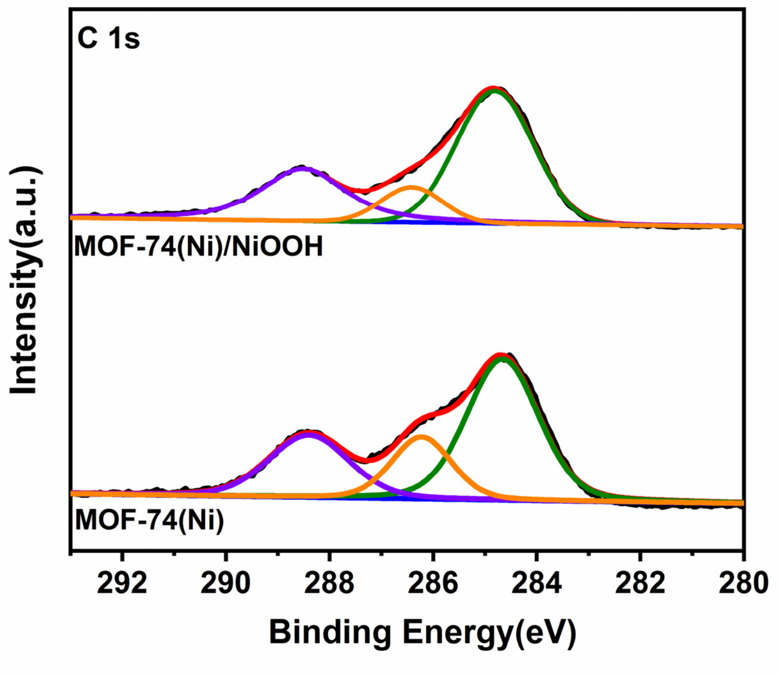

Figure S3. The high-resolution XPS spectra of C1s of MOF-74(Ni) and MOF-74(Ni)/NiOOH.

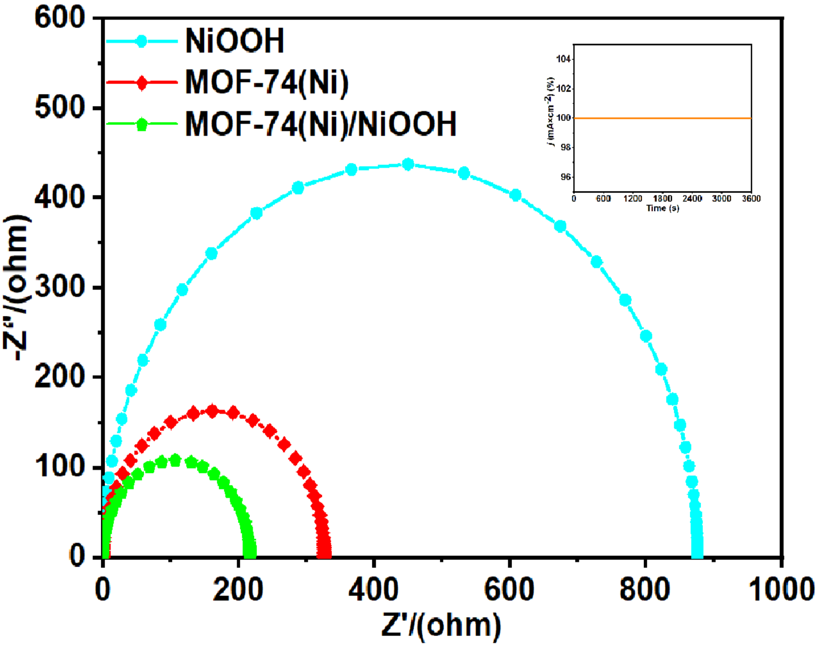

Figure S4. Electrochemical impedance spectra (EIS) of different electrocatalysts.(Inset: Durability measurements for MOF-74(Ni)/NiOOH).

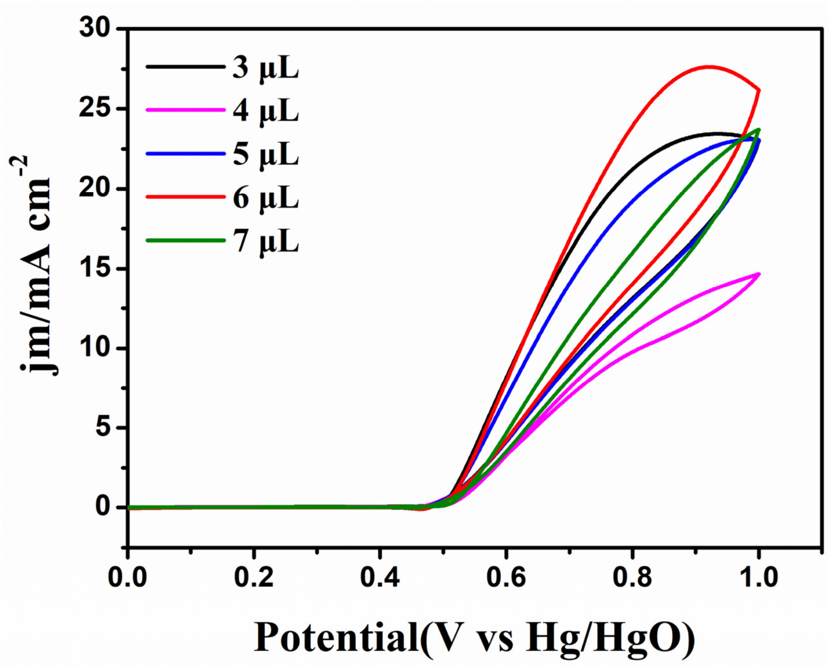

Figure S5. MOR curves of MOF-74(Ni)/NiOOH with different amount operated in 0.1 mol·L^{-1}KOH in presence of 1.0 mol·L^{-1} CH3OH.

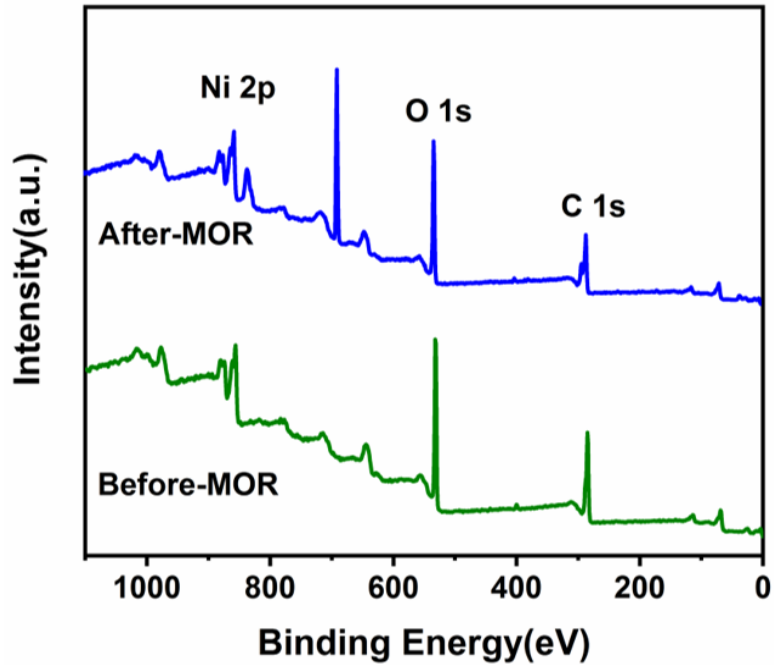

Figure S6. XPS survey spectrum of MOF-74(Ni)/NiOOH before and after MOR.

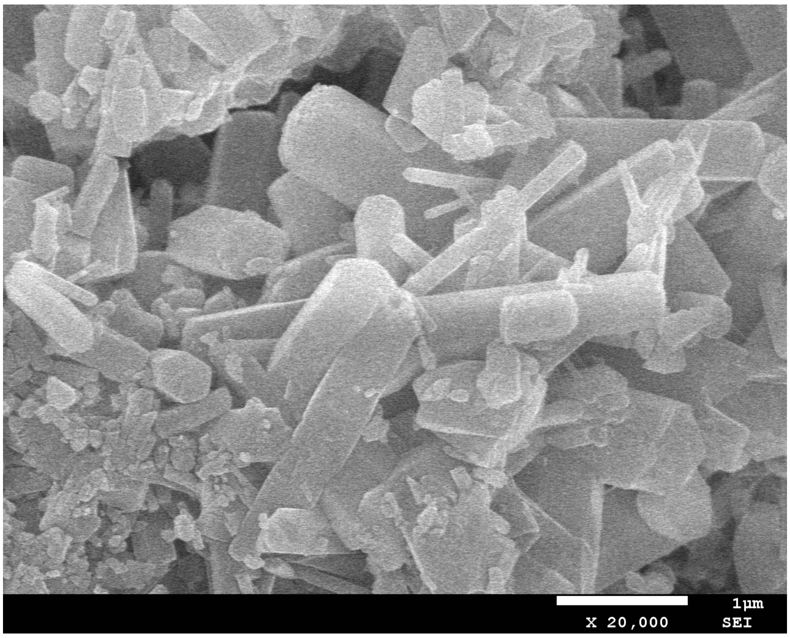

Figure S7.SEM image of MOF-74(Ni)/NiOOH after MOR.
